# Computational identification of harmful mutation regions to the activity of transposable elements

**DOI:** 10.1186/s12864-017-4227-z

**Published:** 2017-11-17

**Authors:** Lingling Jin, Ian McQuillan, Longhai Li

**Affiliations:** 1 Department of Computer Science, University of Saskatchewan, Saskatoon, Canada; 2 Department of Mathematics and Statistics, University of Saskatchewan, Saskatoon, Canada

**Keywords:** Transposable elements, Harmful mutation regions, The human genome, Pearson’s coefficient of correlation, Statistical significance test, Multiple testing correction

## Abstract

**Background:**

Transposable elements (TEs) are interspersed DNA sequences that can move or copy to new positions within a genome. TEs are believed to promote speciation and their activities play a significant role in human disease. In the human genome, the 22 *AluY* and 6 *AluS* TE subfamilies have been the most recently active, and their transposition has been implicated in many inherited human diseases and in various forms of cancer. Therefore, understanding their transposition activity is very important and identifying the factors that affect their transpositional activity is of great interest. Recently, there has been some work done to quantify the activity levels of active *Alu* TEs based on variation in the sequence. Given this activity data, an analysis of TE activity based on the position of mutations is conducted.

**Results:**

A method/simulation is created to computationally predict so-called harmful mutation regions in the consensus sequence of a TE; that is, mutations that occur in these regions decrease the transpositional activity dramatically. The methods are applied to the most active subfamily, *AluY*, to identify the harmful regions, and seven harmful regions are identified within the *AluY* consensus with *q*-values less than 0.05. A supplementary simulation also shows that the identified harmful regions covering the *AluYa5* RNA functional regions are not occurring by chance. This method is then applied to two additional TE families: the *Alu* family and the *L1* family, to computationally detect the harmful regions in these elements.

**Conclusions:**

We use a computational method to identify a set of harmful mutation regions. Mutations within the identified harmful regions decrease the transpositional activity of active elements. The correlation between the mutations within these regions and the transpositional activity of TEs are shown to be statistically significant. Verifications are presented using the activity of *AluY* elements and the secondary structure of the *AluYa5* RNA, providing evidence that the method is successfully identifying harmful mutation regions.

## Background

Transposable elements were first discovered by Barbara McClintock in the 1950s during her studies of maize [1]. They are found in both eukaryotic and prokaryotic organisms, including plants, animals, bacteria, and archaea. Transposable elements were dismissed at one point as being useless, but they are emerging to be thought of as major players in evolution. Additionally, more and more evidence is emerging that active TEs play a significant role in human biology as they create genetic diversity in human populations and can integrate into genes, potentially causing disease.

The proportion of TEs in a genome differs broadly depending on the organism, ranging from 0.3% in *Escherichia coli* to most of the genome (> 80%) in *Zea mays*. In humans, 66–69% of the genome is repetitive or repeat-derived [2], whereas coding sequences comprise less than 5% of the genome. The majority of repeats in human are transposable elements, making up about 45% of the genome [3]. Some TEs have an evolutionary history dating back hundreds of millions of years during which they diversified to share very little sequence homology. Over time, inactivated copies of these TEs have accumulated and now comprise a significant proportion of many genomes, serving as an important opportunity to study molecular evolution. This is because every element in the genome represents a “fossil record” that accumulated mutations independently, meaning that they can be used to study genomic changes both between and within species.

Transposable elements are traditionally classified into two broad categories based on their transposition mechanism and sequence organization [4]. Class I elements are referred to as *retrotransposons* and they have a “copy-and-paste” mechanism that transposes via reverse transcription of an RNA intermediate. The RNA intermediate is first transcribed from a genomic copy, then it is reverse-transcribed back into DNA that is identical to the original DNA by a reverse transcriptase encoded in the TE sequence, and this process produces one new copy in the host DNA [5]. Consequently, retrotransposons can increase the number of copies of TEs, which thereby increases genome size. Class II elements are called *DNA transposons*, and they use a “cut-and-paste” mechanism to move primarily through a DNA-mediated mechanism of excision and insertion.

TEs can be further divided into four types — LTR retrotransposons, LINEs, SINEs, and DNA transposons — on the basis of the structural features of their sequences. Among these types of TEs, non-LTR retrotransposons (LINEs and SINEs) have been major factors of genome evolution by providing diversity and plasticity to the genome [6]. Within each type, TEs are subdivided into families and subfamilies, based on the transposition mechanism, and sequence similarity. For example, *L1*, *L2* are LINEs families, and *Alu*, *SVA* are SINEs families. Furthermore, there are subfamilies *AluY*, *AluJ*, *AluS* of the *Alu* family. TEs are also called *autonomous* or *non-autonomous* based on whether or not they encode the genes used for transposition. Note however that autonomous does not imply that an element is active or functional. A TE can be as *active* if it can transpose either autonomously or non-autonomously.

Typically, the lifespan of one transposable element starts from an activation of the transposon, followed by a rapid burst of activity, while accumulating mutations, followed by the slowing of transpositional activity after additional mutations. The transposon then ebbs further until it becomes inactive. The inactive elements, referred to as *fossil transposable elements*, become relics and can get interrupted by the transpositions of other active elements [7]. Active elements comprise only a tiny proportion of the TE content of the genomes of most organisms. The genomes of eukaryotes are filled with thousands of copies of the remnants of inactive TEs. For example, there are roughly 50,000 autonomous and 200,000 non-autonomous fossil DNA transposons in the human genome, and none of them are active any more [8].

Consensus repetitive sequences (TEs and other repeats) in eukaryotes have been reconstructed and captured in a database called Repbase Update [9]. Repbase is the primary reference database of TEs used in DNA annotation and analysis.

### Motivations

The genomes of most organisms have only a small proportion of active TEs. The genomes of eukaryotes are filled with thousands of copies of the remnants of inactive TEs. For instance, out of the over 500,000 *L1*s in the human genome, there are only about 100 active copies [10]. A gust of transposition of *L1* and *Alu* elements in the primate lineage occurred about 40 million years ago (MYA), followed by a slowing of transpositional activity since then [11]. Recent evidence indicates that there are 35 to 40 subfamilies of *Alu*, *SVA*, and *L1* elements staying actively mobile in the human genome [6, 12], and all of the active transposable elements comprise less than 0.05% of the nucleotides in the human genome. It has been estimated that active human transposons generate about one insertion for every 10 to 100 live births [13–15]. The rate of *L1* retrotransposition is estimated as 1/140 live births per generation [16], and one new *Alu* insertion is generated for every 20 live human births [15].

*Alu* transposition events can have a major impact on human disease [12], as active TEs can integrate into important genes. In fact, there have been forty-three disease-causing *Alu* insertions identified [17]. In very recent research on Alzheimer’s disease, a molecular mechanism of the Alzheimer’s process was proposed to be caused by the *Alu* elements losing their normal controls as a person ages, causing damage to the normal machinery that supplies energy to brain cells, which can lead to a loss of neurons and dementia [18]. The authors hypothesize that *Alu* insertions in mitochondrial genes can lead to progressive neurological disfunction. Therefore, it is of importance to understand how the activity level of *Alu*s can change based on possible mutations.

**Fig. 1:**
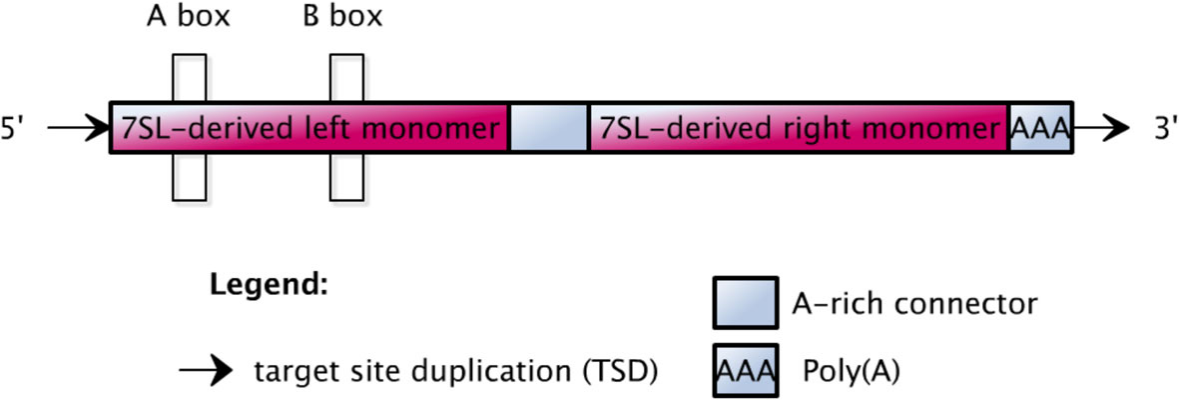
Structure of *Alu* elements

*Alu* elements are approximately 300 base pairs long, and are non-autonomous. They rely upon *L1*-encoded proteins for their own mobilization [19]. *Alu* elements have a dimeric structure of two similar monomers (the left and right arms) that are joined by a linker and terminated with a poly(A) tail [20]. As shown in Fig. 1, the left arm contains weak (but functional) A and B boxes of the RNA polymerase III internal promoter [12].

Different periods of evolutionary history have given rise to different families and subfamilies of *Alu* elements, each containing a small number of active *Alu* elements that serve as the source of subsequent families [8]. According to Repbase Update, there are three *Alu* subfamilies. *AluJ* is the oldest at about 65 million years old, and is thought to be completely inactive [12, 17, 21, 22]. Next, the *AluS* subfamily is the second oldest, as they became active approximately 30 million years ago, and some elements are still active in humans [22, 23]. Lastly, *AluY* is the youngest subfamily, and most elements are currently active [24]. Because there is no specific mechanism for removal of *Alu* insertions, *Alu* evolution is dominated by the accumulation of new *Alu* inserts [8]. These new copies of *Alu* accumulate mutations independently over time.

In order to analyze the transpositional activity of the active *Alu* copies in the human genome, an in vivo plasmid-based mobilization assay was designed in [22] to examine the mobilization capacity of *Alu* copies across the human genome. Generally, the *Alu* retrotransposition was detected on induction by LINE expression vectors. Human HeLa cells were co-transfected with a marked *Alu* element and an expression vector for the human *L1* under the control of the CMV promotor. Cells were amplified and transposition events were detected. This method allows for comparing the relative mobilization efficiencies of varying core elements without changing other factors and eliminating variation of flanking sequences.

Some representative elements were carefully selected from the database of 850,044 full-length human *Alu* copies in [22], in addition to several synthetic older consensus elements that are no longer present in the modern human genome, totalling 89 elements, with 52 *AluY*, 28 *AluS*, and 9 *AluJ*. These elements were then cloned and tested in a mobile assay. From the functional analysis of these *Alu* elements in the mobile assay, the elements that had fewer changes relative to the consensus sequences tended to have the highest levels of activity. Indeed, no elements with more than 10% mutations (at least 28 bp changes) were active [22]. Hence, the amount of sequence variation is an effective factor in altering transpositional activity. However, polymorphic *AluY* copies had higher transpositional activity than randomly chosen *AluY* copies with sequence variation, indicating that some sequence changes are more effective than others in altering activity. Therefore, more analysis is required to understand more precisely what influences TE activity.

A new computational method is developed in this paper to further analyze how the sequence of an element influences its transpositional activity. This method identifies the most critical regions lying within the *AluY* consensus such that mutations have a critical effect in deactivating the elements’ transposition, called “*harmful mutation regions*”. This analysis can be applied to any TE in any organism with experiments akin to those in [22], providing a quantified transposition fraction for each TE.

## Methods

### Materials

In this section, the 52 *AluY* sequences from the experiment in [22] will be analyzed, as the *AluY* family is the youngest *Alu* subfamily with the largest number of active elements. To start, pairwise sequence alignments of the *AluY* consensus sequence (gathered from Repbase Update) versus the *AluY* elements (from the experiment) were calculated, giving pairwise scores for every *AluY* element sequence with the consensus. *percent identity* is used here for pairwise scores.

*AluYa5* elements were used in [22] as a standard for comparing the transposition. Thus, an element is considered more active than *AluYa5* when the cell culture of this element showed greater fluorescence intensity than the cell culture of *AluYa5*, and vice versa. Then, the average *activity fraction* of a TE is defined as a percentage of the fluorescence intensity of the cell culture of this TE over that of *AluYa5* elements. The *Alu* elements can then be categorized by their average activity fraction (which ranges from 0 to 118% of *AluYa5* activity — it can be over 100% if the activity is higher than *AluYa5*). Starting from these activity fractions, all *Alu* elements were organized into four activity level groups as in Definition 1.

#### **Definition 1**

*AluY* elements are grouped into:

- the *inactive group*, that consists of elements with activity fractions that range from 0 to < 5%,
- the *low activity group*, that consists of elements with activity fractions that range from 5 to < 40%,
- the *moderate activity group*, that consists of elements with activity fraction that range from 40 to < 66.6%,
- the *high activity group*, that consists of elements with activity fraction greater than 66.6*%*.

**Fig. 2:**
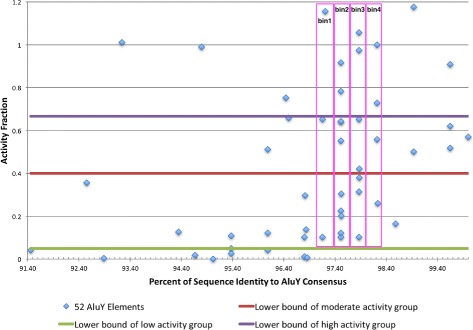
Plot of the 52 *AluY* elements. The plot of the 52 *AluY* elements from [22]. Each element is a point, where the *x*-axis is the percent identity versus the consensus and the *y*-axis is the activity fractions. The elements are partitioned into different groups of activity levels according to the group definitions. Some elements are also grouped into vertical bins for further analysis

The percent identity versus the consensus of all *AluY* elements were plotted against their activity fractions in Fig. 2, where the *x*-axis is the percent identity and the *y*-axis is the activity fraction. Each data point represents one *AluY* element.

The elements are also divided into activity groups as per Definition 1 (i.e. high, moderate, low, and inactive group, as marked in Fig. 2). Although the elements with a higher percent identity tend to have a higher activity level, a linear relationship is not clear. For example, there exist some elements in the high activity group with a low percent identity; conversely, some elements have a higher percent identity but are in the low activity or inactive groups. This leads to the hypothesis that some mutation sites are more affective than others in altering the elements’ transpositional activities. Hence, a computational method is proposed in the next subsections to identify these harmful regions.

### Notations

Some notations need to be provided before describing the computational method.

#### **Definition 2**

For the method, variable names are used so that the method applies in a variety of circumstances. The *total number of elements* in the TE family is denoted by *N*, and the *length of the consensus sequence* is denoted by *L*.

For example, considering the 52 *AluY* elements in [22] with a consensus of 282, then *N*=52 and *L* = 282.

A *window* is a region within the consensus, and is defined by a window size, denoted by *wsize*, and a start position of the window.

Then, a window denoted by *w*_*i*_ is the region of the consensus from the *i*th position to the position *j*=*i*+*wsize*−1.

Hence, the *number of windows*, denoted by *nw*, can be calculated as *nw*=*L*−*wsize*+1.

#### **Definition 3**

*Mutations within the window* *w*_*i*_ of one TE element is defined as the total number of mutations (versus the consensus) of this element lying within the window, denoted by *m*_*i*_.

For example, for an element with mutations at positions 2,3,7,15,80,224 in the consensus, with *wsize*=10, then *m*_1_=3 (number of mutations in the window from position 1 to 10), and *m*_10_=1 (number of mutations in the window from position 10 to 19).

#### **Definition 4**

For every element, every window from the beginning to the end of the consensus is considered, to generate a vector of mutations in all windows for this element. Mutations in every window of every element can be represented as a *mutation matrix*, denoted as *M*(*N*×*nw*).

1 $$  M(N\times nw)= \left[\begin{array}{lllll} m_{11} & m_{12} & m_{13} & \ldots & m_{1nw} \\ m_{21} & m_{22} & m_{23} & \ldots & m_{2nw} \\ \vdots & \vdots & \vdots & \vdots & \vdots \\ m_{N1} & m_{N2} & m_{N3} & \ldots & m_{Nnw} \\ \end{array}\right]  $$

**Fig. 3:**
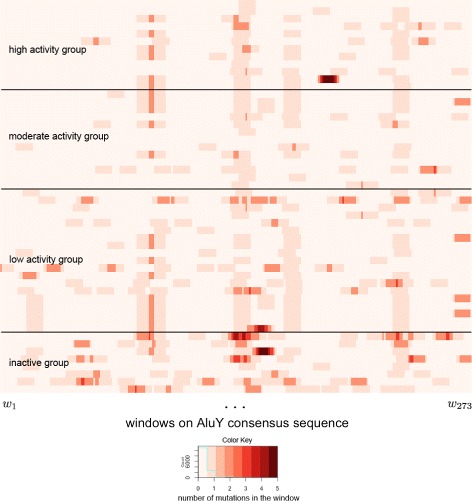
The number of mutations in each window of the *AluY* elements. The number of mutations versus the consensus of every *AluY* elements of Fig. 2 in each window with *wsize*=10. TEs are sorted in descending order by their activity fractions from the top to the bottom of the chart

Taking the example of the *AluY* elements in [22], there are *N*=52 rows and *nw*=273 (where *L*=282,*wsize*=10) columns in the matrix. The mutation matrix of the *AluY* elements, representing the mutations of each element in each window, is shown as a heat map in Fig. 3, where the windows are shown in the *x*-axis and the *AluY* TEs sorted by descending activity fractions are shown in the *y*-axis. The activity groups are also marked with black lines in the figure.

It is easy to see from Fig. 3 that certain windows are darker than others, which provides visual evidence that certain regions in the sequence tend to have more mutations. However, the heat map alone does not help indicate which mutations are correlated with a change in transpositional activity, nor does it show how they are related. By using correlation analysis method below, it will be shown that mutations in certain windows are indeed harmful to elements’ activities.

### Pearson’s coefficient of correlation and multiple test correction

In this section, a method is proposed to identify the harmful regions in an active TE by using the Pearson’s coefficient of correlation. The *Pearson’s coefficient of correlation* (denoted by *ρ*) is used to measure the linear correlation between two variables *X* and *Y*. The result ranges from -1 to 1, with 1 indicating total positive correlation, 0 indicating no correlation, and -1 indicating total negative correlation. It is defined as the covariance of the two variables, *cov*(*X,Y*), divided by the standard deviation of *X*, *σ*_*X*_, multiplied by the standard deviation of *Y*, *σ*_*Y*_,

2 $$ \begin{aligned}  \rho = \frac{cov(X,Y)}{\sigma_{X}\sigma_{Y}}. \end{aligned}  $$

The Pearson’s correlation coefficient is a model-free method, as it shows the nature of the data without being built on an existing model. Indeed, in model-based methods, if data does not fit the model perfectly, results can be misleading.

**Fig. 4:**
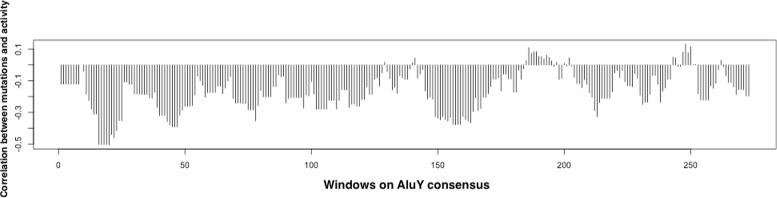
The Pearson’s coefficients of correlation between the number of mutations in each window and the activity fractions of the *AluY* elements. The *x*-axis gives the windows in order on the *AluY* consensus

For each window in the *AluY* consensus, the variable *X* is the number of mutations in each window of each *AluY* element, and the variable *Y* is the activity fraction of each *AluY* element. The Pearson’s coefficient of correlation was calculated by comparing *X* against Y, using the correlation function cor in the R Language. The observed correlations from the data in the experiment in [22] are calculated and denoted by

$$\rho_{obs} = (\rho_{1}, \rho_{2}, \ldots, \rho_{nw}), $$

as shown in Fig. 4. It can be seen that mutations occurring in most windows have negative correlation with the transpositional activity. As negative correlation indicates that the TE activity decreases as the number of mutations in a window increases, the mutations in these windows are harmful to TE activity. However, to evaluate whether these negative correlations arise by randomness/chance, a statistical significance test is used. The *p*-value measures the probability that more negative correlations than what was observed in the data set can be caused solely by chance. This is a measure of significance in terms of the false positive rate [25].

To correct for multiple comparison bias caused by the large number of windows, a *q*-value is also reported. A *q*-value is a similar measurement to a *p*-value. It is a quantity for convenience of reporting the “false discovery rate” (FDR) [26]. The false positive rate and FDR are defined differently — given a rule for calling features significant, the false positive rate is the rate that truly null features are called significant, while the FDR is the rate at which significant features are truly null [27]. As an example, a false positive rate of 5% in a study means that 5% of the truly null features are called significant on average, while a FDR of 5% indicates that 5% of all features that are called significant are truly null. In general, the FDR is a sensible measure capturing the balance between the number of true positives and false positives. Multiple testing correction will be performed using the qvalue package [28] under Bioconductor in the R Language.

## Results

In order to investigate the relationships between the transpositional activity and the mutations of a TE, a null hypothesis is proposed as “**mutations in a window are not negatively related (or undifferentiated) to the activity of the TE”**. To test the hypothesis, a statistical simulation is used to generate random data as elaborated in the steps below. The framework of the simulation is a general statistical technique for hypothesis testing.

**Fig. 5:**
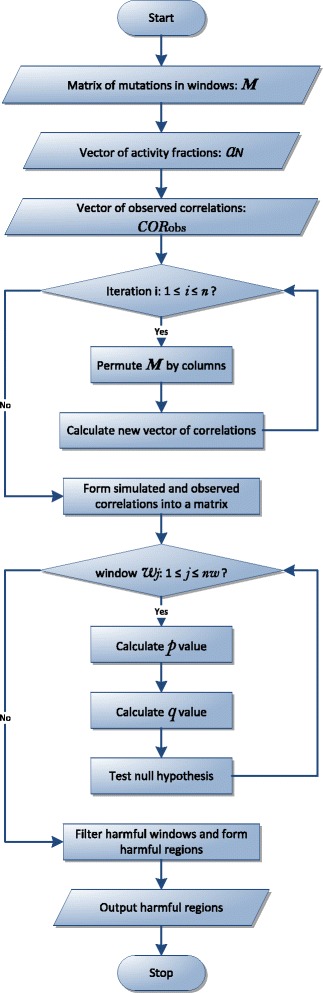
The flow chart of the computational method

Given a mutation matrix, *M*(*N*×*nw*), as described in Eq. (1), the activity fractions vector of the *N* elements, *α*_*N*_, and the observed correlations *ρ*_*obs*_, perform the following operations (also depicted with the flow chart in Fig. 5).

Step 1: generate simulated correlations.

Given the number of iterations as *n* (eg. *n*=1000), for each iteration denoted by *i*,

1. permute *M* by columns as *M* ^*i*^;
2. calculate correlations between *M* ^*i*^ and *α*_*N*_. The correlations for each window for this iterations is denoted by *ρ*_(*i*,1)_,*ρ*_(*i*,2)_,…,*ρ*_(*i,nw*)_.

Step 2: form simulated and observed correlations into a matrix.

After the *n* iterations, there are *n* simulated correlations for each window. The simulated correlations along with the observed correlations are formed into a matrix and summarized in Table 1.

**Table 1:** Simulated and observed correlations between mutations and transpositional activity

Iteration	*w*_1_	*w*_2_	…	*w*_*j*_	…	*w*_*nw*_
1	*ρ*_(1,1)_	*ρ*_(1,2)_	…	*ρ*_(1,*j*)_	…	*ρ*_(1,*nw*)_
2	*ρ*_(2,1)_	*ρ*_(2,2)_	…	*ρ*_(2,*j*)_	…	*ρ*_(2,*nw*)_
⋮	⋮	⋮	⋮	⋮	⋮	⋮
i	*ρ*_(*i*,1)_	*ρ*_(*i*,2)_	…	*ρ*_(*i,j*)_	…	*ρ*_(*i,nw*)_
⋮	⋮	⋮	⋮	⋮	⋮	⋮
*n*	*ρ*_(*n*,1)_	*ρ*_(*n*,2)_	…	*ρ*_(*n,j*)_	…	*ρ*_(*n,nw*)_
observed correlation (*ρ*_*obs*_)	*ρ*_1_	*ρ*_2_	…	*ρ*_*j*_	…	*ρ*_*nw*_
*p*-value	*p*_1_	*p*_2_	…	*p*_*j*_	…	*p*_*nw*_
*q*-value	*q*_1_	*q*_2_	…	*q*_*j*_	…	*q*_*nw*_

Step 3: calculate *p*-values for each window.

For each column *w*_*j*_ (1≤*j*≤*nw*) in Table 1, calculate a *p*-value of *ρ*_*j*_ in the distribution of *ρ*_(*i,j*)_(1≤*i*≤*n*), which is *p*_*j*_=*P*(*ρ*_(*i,j*)_≤*ρ*_*j*_), where 1≤*j*≤*nw*.

Step 4: calculate *q*-values for each window.

After the *p*-values are calculated for each window, estimate the *q*-values of each window, *q*_1_,*q*_2_,…,*q*_*nw*_ using the function qvalue in the R Language.

Step 5: test the null hypothesis for each window.

For each window *w*_*j*_ (1≤*j*≤*nw*), compare its *q*-value, *q*_*j*_, to a confident threshold *λ* (eg. *λ*=0.05). If *q*_*j*_<*λ*, we can reject the null hypothesis. If the null hypothesis is rejected, then the window *w*_*j*_ is harmful, and the sites in the window are harmful sites.

Step 6: filter out all windows that are harmful and form overall harmful regions.

The example below illustrates how to test if a specific window, *w*_20_, is considered to be a harmful window by comparing the observed correlation and simulated correlations between the elements’ transpositional activity and mutations, by using the method above.

### **Example 1**

**Fig. 6:**
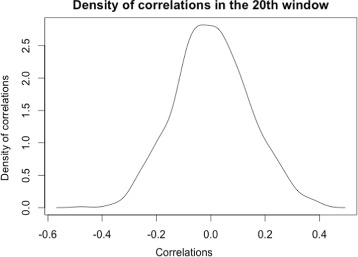
The density plot of correlations. The density plot of the simulated correlations in the window between position 20 and 29

Consider in particular an example with a window size *wsize*=10, the number of iterations *n*=10,000, and consider window *w*_20_ (between positions 20 and 29). Given the matrix of mutations, *M*, the method calculates the observed correlation of *w*_20_ by comparing the number of mutations in the 20th window, *M*[,20] (the 20th column of the matrix), and the elements’ activity fractions vector, *α*_*N*_. The observed correlation is calculated to be *ρ*_20_=−0.5059255. Then the following steps are performed:

Step 1: permute *M*[,20]*n* times and calculate the correlation for every permutation, denoted by *ρ*_(1,20)_,*ρ*_(2,20)_,…,*ρ*_(*n*,20)_. The density plot of the simulated correlations *ρ*_(1,20)_,*ρ*_(2,20)_,…,*ρ*_(*n*,20)_ is shown in Fig. 6.

Step 2: calculate the *p*-value of the observed correlation in the distribution: *p*_20_=*P*(*ρ*_(*i*,20)_≤*ρ*_20_)<0.00001. Using the same method, the *p*-values of all windows can be calculated.

Step 3: perform a multiple test correction to calculate the *q*-values. The *q*-value of the window in this example is calculated as *q*_20_<0.00001.

Step 4: given the confident threshold *λ*=0.05, we can reject the null hypothesis. Hence, the window *w*_20_ is considered as a harmful window, which means that mutations occurring within this window are more affective to the transpositional activity of the *AluY* elements.

**Fig. 7:**
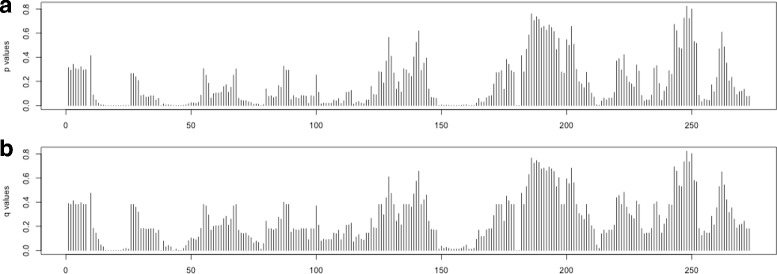
The *p*-values in **a**, and *q*-values in **b** of the *AluY* elements. The *x*-axis gives the windows in order on the *AluY* consensus

Using this method, the *p*-value and *q*-value are calculated for every window in the *AluY* consensus and the results are shown in Fig. 7 a and b respectively. Given a confidential threshold *λ*=0.05, a window in the *AluY* consensus is identified as a harmful window if and only if its *q*-value ≤*λ*. The harmful windows that are overlapped are classified into harmful regions as listed in Table 2. With *λ*=0.05, the identified harmful regions in Table 2 cover 34.5% of the total length of *AluY* consensus sequence. Next, these computationally identified regions will be verified to be harmful to the activity of *AluY* elements.

**Table 2:** The harmful mutation regions in *AluY* elements calculated from correlation analysis (*λ*=0.05)

Region ID	RegionStart	RegionEnd	Average *q*-value
1	14	34	0.0101
2	38	57	0.0183
3	78	87	<0.0001
4	149	172	0.0178
5	180	190	<0.0001
6	212	222	0.0232

## Verifications

In this subsection, the harmful regions predicted in Table 2 will be verified in two different ways. First, the *AluY* elements with a similar percent identity versus the consensus are examined to see if having various activity is due to whether or not mutations occurred in harmful regions. Second, a possible reason for the harmful regions affecting transpositional activity is because the harmful regions overlap with the functional sites of the *AluYa5* RNA that are important for the transposition of the elements.

### Verification by activities of *AluY* elements

Each of the *AluY* elements from [22] are compared to the consensus sequence. The relationship between the percent identity of these elements and their transpositional activity levels is plotted in Fig. 2. By grouping elements with similar percent identity into vertical bins, as marked in the figure, it is evident that the activity levels of the elements in the same bin vary considerably. As an example, the elements in bin #1 all have approximately 97% percent identity with the consensus, but their activity levels range from 1*%* to 106*%* (in comparison to the activity of *AluYa5*).

One possibility for this difference is that some mutations occur within the harmful regions, which decreases their activities dramatically. Therefore, all mutations in the high activity group are classified as “neutral sites”, as activity levels remain high despite the mutations. Table 3 lists the percentage of mutations that occurred in both the harmful region and neutral region for each activity group in the bins in Fig. 2. Notice that, in the low activity groups of each bin, there are more mutations in the harmful region compared to other activity groups. Moreover, none of the mutations in the high activity groups falls into the harmful regions. Therefore, it is reasonable that the mutations that occurred in the identified harmful regions may indeed cause the low activity levels of these elements.

**Table 3:** The percentage of mutations grouped by bins marked on Fig. 2 over the total number of mutations in each group

	Activity group	Harmful regions	Neutral sites
bin 1	low activity	13%	63%
	moderate activity	13%	63%
	high activity	0%	100%
bin 2	low activity	0%	0%
	moderate activity	0%	5%
	high activity	0%	0%
bin 3	low activity	6%	0%
	moderate activity	0%	11%
	high activity	0%	8%
bin 4	low activity	0%	33%
	moderate activity	40%	20%
	high activity	0%	10%

### Verification by *AluYa5* RNA secondary structure

**Fig. 8:**
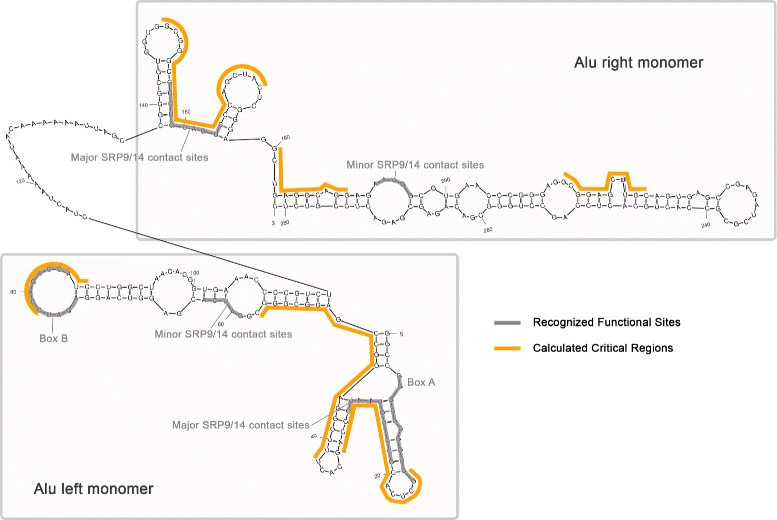
The secondary structure of an *AluYa5* RNA. The SRP contact sites and the A and B boxes are marked in grey. The harmful regions identified in Table 2 are marked in yellow along the structure as indicated in the legend

As described previously, *Alu* elements have left and right arms, and the left arm contains A and B boxes of the RNA polymerase III internal promoter. Figure 8 shows the secondary structure of the *AluYa5* RNA as predicted by Mfold [29] (a secondary structure prediction program) based on previously determined secondary structure in [20, 30]. It is known that SRP9/14 binding is necessary for efficient *Alu* mobilization, and the left *Alu* monomer binding to SRP9/14 is more important for mobilization than the right *Alu* monomer binding [22]. In Fig. 8, both the major and the minor SRP contact sites, and the A and B boxes, are marked on the secondary structure in grey; the identified harmful regions in Table 2 are marked in yellow. As shown in Fig. 8, the harmful regions “cover” the two major SRP contact sites and the B box very well, and there are three additional unknown regions that are also recognized as harmful. The unknown regions might have some interesting unknown function.

Next, it will be shown via simulation that the identified harmful regions do not cover the functional regions totally randomly (by chance). The *coverage of harmful regions* is defined to be the percentage of the overlapped number of positions between harmful regions (marked in yellow) and functional regions (marked in grey) divided by the total number of positions in the functional regions (marked in grey). The simulation compares the coverage of the harmful regions and that of randomly generated regions as follows: given the lengths and positions of functional regions (*nf* as number of functional regions), the lengths and positions of harmful regions (*nh* as number of harmful regions), and the number of trials as *n*,

1. calculate the coverage of harmful regions
  $$ Cov_{\text{harmR}}. $$
2. For every iteration *i*, where 1≤*i*≤*n*,
  - randomly generate *nh* regions with the same lengths as the harmful regions identified as shown in Table 2, and the algorithm makes sure that these regions do not overlap with each other;
  - calculate the coverage of randomly generated regions in this iteration, denoted by $\phantom {\dot {i}\!}Cov_{\text {randR}^{i}}$.
3. After *n* iterations, there are *n* generated coverages, denoted by
  $$Cov_{{\text{randR}^{1}}}, Cov_{{\text{randR}^{2}}}, \ldots, Cov_{{\text{randR}^{n}}}. $$
4. Calculate the probability where the coverage of harmful regions is less than the coverage of random regions as
  $$P(Cov_{\text{harmR}} < Cov_{\text{randR}}). $$

**Fig. 9:**
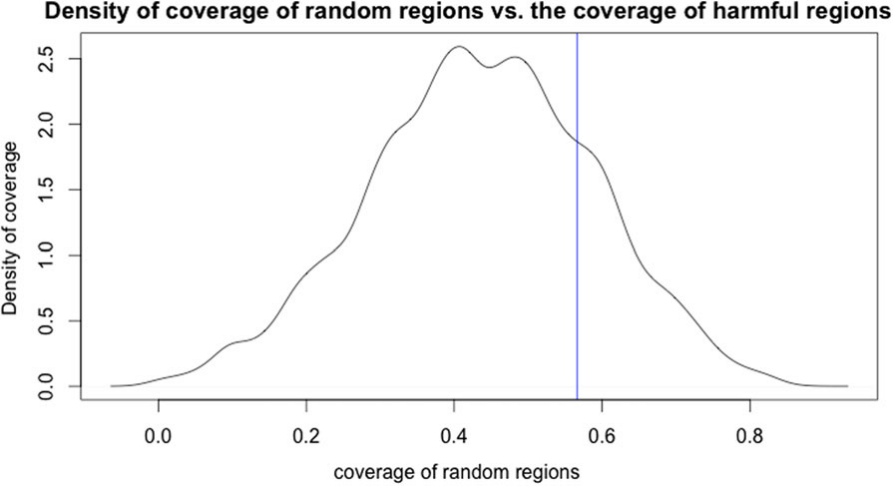
The density plot of coverage. The density of the coverage of random generated regions. The blue vertical line is the coverage of the harmful regions

Executing the simulation with this method on the *AluY* harmful regions calculated in Table 2 for *n*=10,000 iterations, Fig. 9 shows the density of the empirical distribution of the coverage of random regions, and the blue line on the figure shows the coverage of the harmful regions in Table 2.

The probability where the coverage of harmful regions is less than the coverage of random regions is calculated as *P*(*Cov*_harmR_<*Cov*_randR_)=22*%*; that is, 78% of randomly generated regions have less coverage than the harmful regions identified by our method. Therefore, the harmful regions cover the *AluY* functional regions and we conclude that this coverage is likely not by chance.

## Additional case studies

The computational method proposed to calculate the harmful mutation regions of TEs was applied to a specific TE family (the *AluY* subfamily) where the transpositional activity fractions of the elements in this family were quantified in [22]. The predicted regions of the *AluY* elements using this method were verified using both the activities of *AluY* elements and the *AluYa*5 RNA secondary structure, which also supports the correctness of the computational method proposed. In this section, this method will be applied to two other cases — the *Alu* family generally and the *LINE-1 (L1)* family, to identify the harmful mutation regions lying within their consensus sequences.

### The *Alu* family

The work in [22] has systematically tested 89 representatives from many *Alu* families and also subfamilies, and all the *AluY* elements have been examined in previous sections. In this subsection, the computational method will be applied to a bigger set of elements of the *Alu* family, including 9 *AluJ*, 28 *AluS*, and 52 *AluY*, where their activity fractions are quantified in [22].

**Fig. 10:**
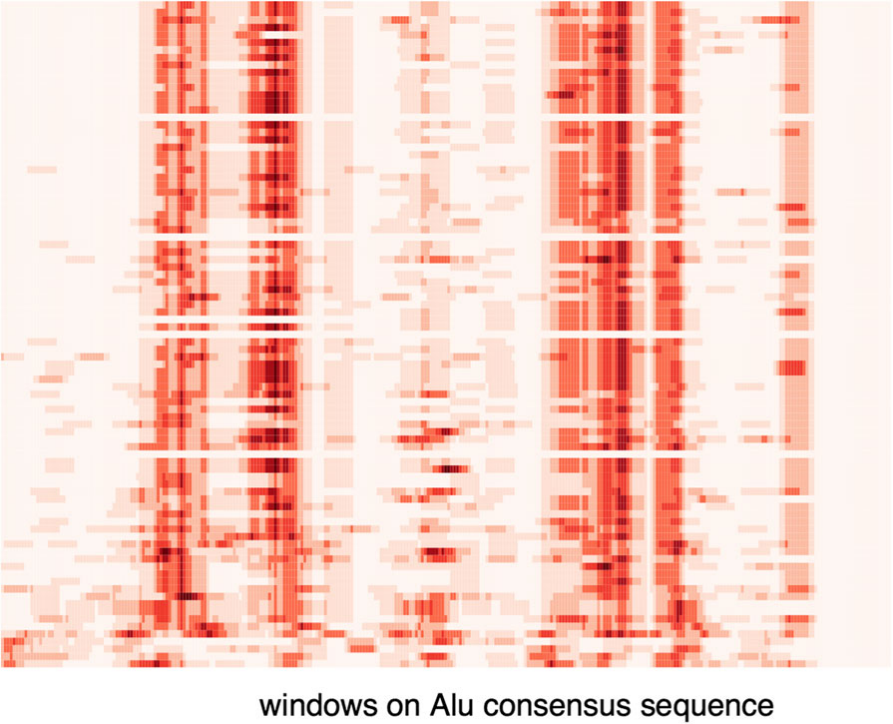
The number of mutations in each window of the *Alu* elements. The number of mutations of the *Alu* elements in each window on the *Alu* consensus (*wsize*=10). The TEs are sorted by their activity fractions in descending order from the top to the bottom of the chart

**Fig. 11:**
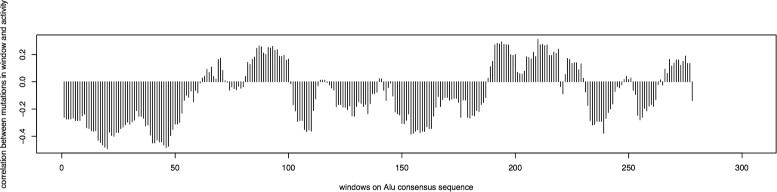
The Pearson’s coefficients of correlation between the number of mutations in each window and the activity fractions of the *Alu* elements. The *x*-axis gives the windows in order on the *Alu* consensus

There are a total of 89 elements (*N*=89) and the length of the *Alu* consensus is *L*=312. First, pairwise sequence alignment of each of the *NAlu* elements is performed against the *Alu* consensus sequence from Repbase Update to get the mutation data for each element. Given the window size as *wsize*=10, calculate a mutation matrix, *M*(*N*×*nw*), as in Eq. 1, where *nw*=*L*−*wsize*+1. This mutation matrix, representing the number of mutations in each window, is plotted in the heat map as shown in Fig. 10. The observed Pearson’s coefficient of correlation between the mutations in windows and the activities of the *Alu* elements are calculated using Eq. 2 and is shown in Fig. 11.

**Fig. 12:**
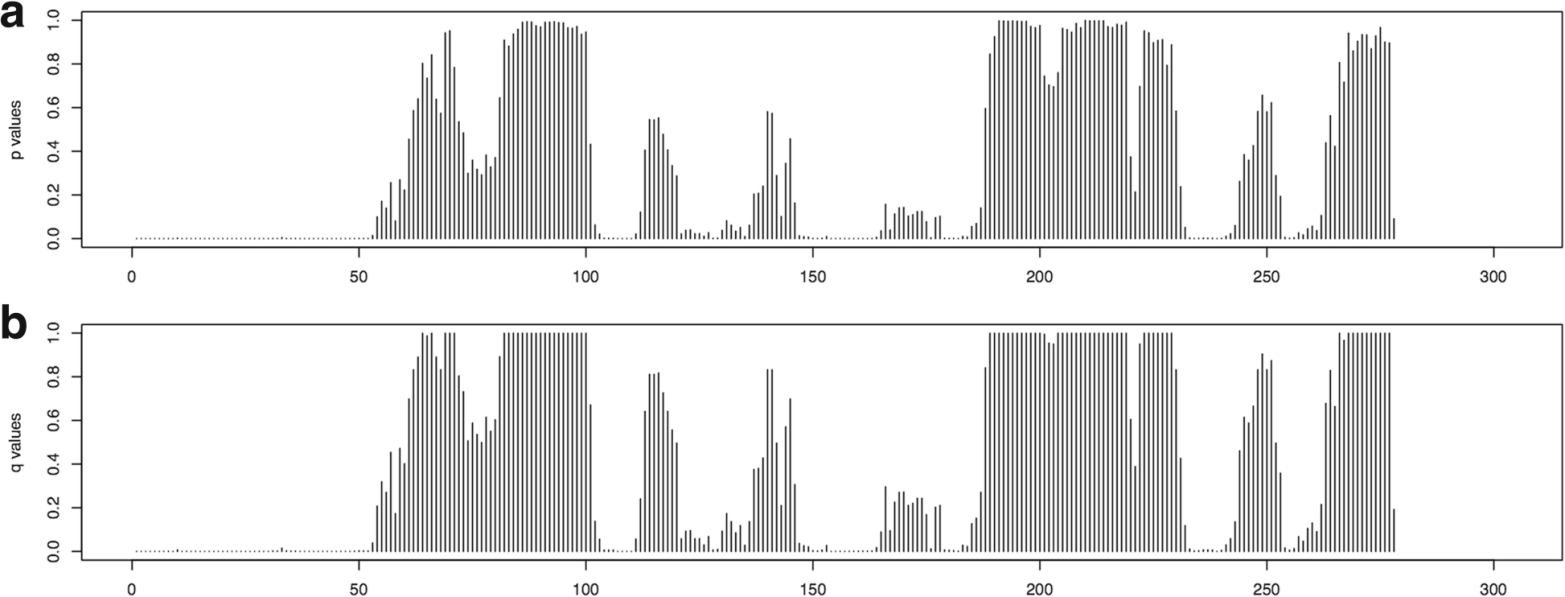
The *p*-values in **a**, and *q*-values in **b** of the *Alu* elements. The *x*-axis gives the windows in order on the *Alu* consensus

Then the steps in the computational method are followed to perform the statistical significance tests on the *Alu* data for *n*=10,000, and the simulated correlations are calculated. The *p*-value and *q*-value are calculated for each window. The results are shown in Fig. 12. Finally, the harmful regions in the *Alu* elements are calculated and listed in Table 4. In summary, the total length of the harmful mutation regions is 171 bp, which is 54.81% of the *Alu* consensus.

**Table 4:** The harmful mutation regions in *Alu* elements calculated from correlation analysis (*λ*=0.05)

Region ID	RegionStart	RegionEnd	Average *q*-value
1	1	62	0.0010
2	104	119	0.0015
3	126	144	0.0181
4	147	173	0.0078
5	176	193	0.0102
6	233	250	0.0066
7	254	266	0.0176

### The *L1* family

*L1* elements make up 17% of the human genome [31]. An active *L1* is about 6 kb in length, and it has been estimated that an average diploid human genome contains approximately 80–100 active *L1s* [32]. In the work of [32], 82 *L1* elements were cloned and each assayed for its ability to retrotranspose in cultured cells. These elements were then compared with the *L*1_*RP*_ element to get their quantified retrotranspositional activity fractions in a similar fashion to [22].

Among the 82 *L1* elements in [32], *N*=77 were retrieved where both their sequences and activity fractions were available. The length of the *L1* consensus sequence (accession no. L19092.1) is 6053 bp (*L*=6053). Using the computational method, a mutation matrix *M*(*N*×*nw*) is generated, and the observed Pearson’s coefficient of correlation between the mutations in windows and the activities of the *L1* elements are calculated using Eq. 2. Then the steps in the computational method are followed to perform the statistical significance tests on the *L1* data for *n*=10,000, and the simulated correlations are calculated. The *p*-value and *q*-value for each window are estimated, which gives the harmful regions in the *L1* elements. There are 201 harmful regions calculated from *N*=77*L1* elements, and the total length of these regions is 3500 bp in total, which covers 57.82% of the *L1* consensus sequence.

**Fig. 13:**
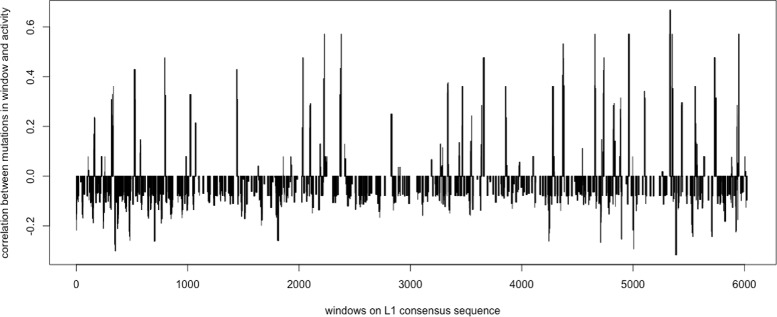
The Pearson’s coefficients of correlation between the number of mutations in each window and the activity fractions of the *L1* elements. The *x*-axis gives the windows in order on the *L1* consensus

**Fig. 14:**
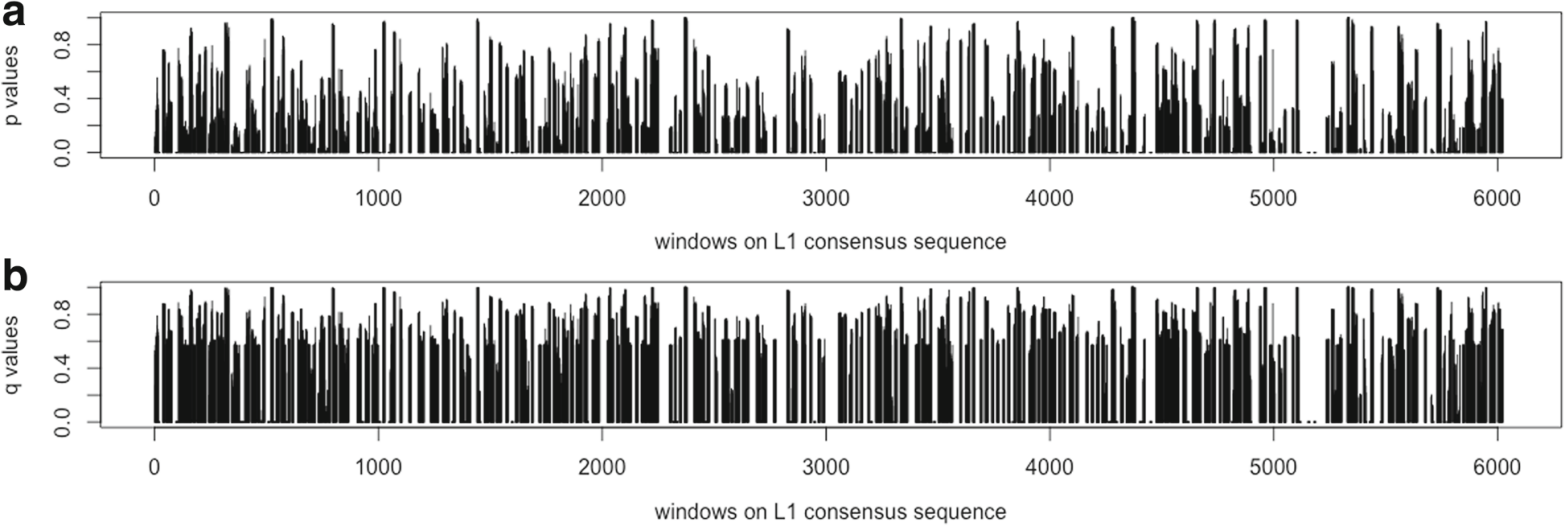
The *p*-values in **a**, and *q*-values in **b** of the *L1* elements. The *x*-axis gives the windows in order on the *L1* consensus

Notice that a large number (38 out of 77) of the *L1* elements have an activity fraction of 0%, and many (25 out of 77) are inactive with activity fractions between 0 to 5%. Due to the large number of elements being inactive (more than 80% of the total number of elements), the effects to the negative correlations between the mutations in these elements and the transpositional activity are biased. Therefore, the same calculation is performed again to only include the elements with non-zero activity fractions (*N*=39). The observed Pearson’s coefficient of correlation between the mutations in windows and the activities of the *L1* elements are calculated using Eq. 2 and shown in Fig. 13. The *p*-value and *q*-value for each window are shown in Fig. 14, and the predicted harmful regions with *λ*=0.01 are listed in Table 5. The total length of these regions is 894 bp, which covers 14.77% of the *L1* consensus.

**Table 5:** The harmful mutation regions in *L1* elements calculated from correlation analysis (*λ*=0.01)

Region ID	RegionStart	RegionEnd	Average *q*-value
1	19	31	< 0.0001
2	94	112	< 0.0001
3	182	191	< 0.0001
4	314	323	< 0.0001
5	353	362	< 0.0001
6	364	375	< 0.0001
7	381	407	< 0.0001
8	474	491	0.0041
9	505	523	< 0.0001
10	530	547	< 0.0001
11	588	603	< 0.0001
12	661	671	< 0.0001
13	698	707	< 0.0001
14	854	864	< 0.0001
15	925	943	< 0.0001
16	1000	1020	< 0.0001
17	1046	1061	< 0.0001
18	1328	1342	< 0.0001
19	1386	1398	< 0.0001
20	1455	1467	< 0.0001
21	1508	1517	< 0.0001
22	1594	1612	< 0.0001
23	1935	1947	< 0.0001
24	2095	2104	< 0.0001
25	2315	2330	< 0.0001
26	2332	2348	< 0.0001
27	2460	2478	< 0.0001
28	2547	2556	< 0.0001
29	2591	2600	< 0.0001
30	2710	2722	< 0.0001
31	2838	2855	< 0.0001
32	2889	2899	< 0.0001
33	2945	2963	< 0.0001
34	2983	2992	< 0.0001
35	3147	3162	< 0.0001
36	3198	3216	< 0.0001
37	3247	3256	< 0.0001
38	3299	3310	< 0.0001
39	3330	3339	< 0.0001
40	3421	3431	< 0.0001
41	3479	3497	< 0.0001
42	3822	3846	< 0.0001
43	3869	3887	< 0.0001
44	4262	4283	< 0.0001
45	4295	4311	< 0.0001
46	4340	4349	< 0.0001
47	4399	4424	< 0.0001
48	4446	4464	< 0.0001
49	4613	4631	< 0.0001
50	4676	4685	< 0.0001
51	4812	4827	< 0.0001
52	4899	4910	< 0.0001
53	5114	5131	< 0.0001
54	5152	5170	< 0.0001
55	5179	5197	< 0.0001
56	5269	5279	< 0.0001
57	5413	5424	< 0.0001
58	5426	5441	< 0.0001
59	5476	5488	< 0.0001
60	5586	5596	< 0.0001
61	5713	5724	< 0.0001
62	5756	5765	< 0.0001
63	5773	5787	< 0.0001
64	5816	5829	< 0.0001

### Discussions

The computational method was inspired by the observation on Fig. 4 of the *AluY* family that mutations occurring in most of the windows have negative correlation with the transpositional activity. In contrast, in Fig. 11 of the *Alu* family and Fig. 13 of the *L*1 family, there are a number of windows that have positive correlation with the transpositional activity. A positive correlation indicates that the TE transpositional activity increases as the number of mutations in a window increases. This might be because of the selection of the consensus sequence, as the mutations are calculated based on the consensus sequence which is assumed to be a “representative” element in that family, and the mutations in younger elements with higher activity relative to the consensus may seem to “increase” the elements’ activity. Furthermore, as was previously mentioned, there might be many factors altering elements’ activities simultaneously and mutations are only one factor among them. Thus, the reasons that some mutations have positive correlations to transpositional activity might be caused by a combination of other unknown factors.

## Conclusions

In this paper, major factors that affect the transpositional activity of TEs is discussed. A computational method is developed to identify specific regions where mutations harm activity, called harmful regions, using correlation analysis and statistical significance tests. The harmful regions are that verified by examining elements with the very similar percent identity but usually different transpositional activity. Moreover, the identified harmful regions were shown to “cover” the *AluY* SRP major binding sites, which is indeed important for the *AluY* element to bind to SRP9/14 for its transposition, also supporting the fact that these regions are important in the transposition of active elements. Three additional harmful regions were also identified. The computational method is then applied to a bigger set of elements of the *Alu* family, and then to the *L1* family to identify their harmful mutation regions. To the best of our knowledge, this is the only work that computationally identifies regions that significantly affect transpositional activity, and there has not been any other studies involving a similar data analysis. However, the role of other factors influencing activity is still unknown. The method was only applied to both the *Alu* and *L1* families in the human genome, as they are highly active in the human genome, and the data of activity levels exist. However, the technique can be easily applied to other families of TEs and other organisms once activity levels and sequence data are determined.
